# Feasibility of Golden Angle Spiral Real-Time Phase Contrast MRI at 0.55T: A Single-Center Prospective Study

**DOI:** 10.3390/bioengineering13020166

**Published:** 2026-01-29

**Authors:** Salman Pervaiz, Chong Chen, Yingmin Liu, Katherine Binzel, Kelvin Chow, Rizwan Ahmad, Yuchi Han, Orlando P. Simonetti, Ning Jin, Juliet Varghese

**Affiliations:** 1 Dorothy M. Davis Heart and Lung Research Institute, The Ohio State University, Columbus, OH 43210, USA; 2 Division of Cardiovascular Medicine, Department of Internal Medicine, The Ohio State University, Columbus, OH 43210, USA; 3 Department of Radiology, The Ohio State University, Columbus, OH 43210, USA; 4 Cardiovascular MR R&D, Siemens Healthcare Ltd., Edmonton, AB T6B 2N7, Canada; 5 Department of Biomedical Engineering, The Ohio State University, Columbus, OH 43210, USA; 6 Cardiovascular MR R&D, Siemens Medical Solutions USA Inc., Malvern, PA 19355, USA; ning.jin@siemens-healthineers.com

**Keywords:** real-time MRI, phase-contrast, flow imaging, compressed sensing, spiral acquisition, mid field

## Abstract

**Background**: Real-time phase-contrast magnetic resonance (RT-PCMR) imaging allows free-breathing assessment of blood flow across cardiac valves and vessels. However, the feasibility of free-breathing RT-PCMR on a mid-field (0.55T) MRI system has yet to be established. **Aim:** The primary objective of this study was to implement a RT-PCMR sequence using a dual-density golden-angle spiral readout with SENSE-based compressed sensing (CS) reconstruction on a 0.55T MRI system. The secondary objective was to evaluate the feasibility of this approach in an adult cohort comprising healthy volunteers and patients with cardiovascular disease. **Materials and Methods:** Data from 33 participants were included in the flow quantification analysis (healthy volunteers: *n* = 17, 9 females, mean age 30.4 ± 14.6 years; patients: *n* = 16, 11 females, mean age 45.9 ± 17.4 years), with breath-held (BH) segmented Cartesian PCMR used as the reference standard. **Results:** In volunteers, RT-PCMR showed good agreement for net flow, peak flow rate, and pulmonary–systemic flow ratio (Qp/Qs), without significant bias (*p* > 0.05) and slightly underestimated peak velocity [7.9% in the aorta and 8.6% in the main pulmonary artery (MPA)]. In patients, RT-PCMR slightly underestimated peak flow rate (aorta, 6.2%; MPA; 4.6%) and peak velocity (aorta,12.7%; MPA, 10.4%). A sub-analysis of six patients scanned at both 0.55T and 3T showed close agreement between field strengths. **Conclusions:** These results demonstrate the feasibility of our RT-PCMR sequence on a commercial 0.55T system.

## 1. Introduction

Cardiac magnetic resonance (CMR) imaging has emerged as a pivotal diagnostic tool for various cardiovascular pathologies such as congenital anomalies, valvular disorders, cardiomyopathies, and ischemic heart disease [1]. Higher field (≥1.5T) systems are currently the standard across various clinical settings due to the inherent benefits of higher signal-to-noise ratio (SNR), enabling improved spatiotemporal resolution and efficient imaging speeds compared to lower field strength systems. However, the unaffordability of high-field scanners is a major constraint in resource-limited settings. Conversely, mid-field scanners may provide a cost-effective and reliable solution, facilitating broader accessibility to CMR technology [2,3]. The recent use of advanced image acquisition and reconstruction techniques on contemporary mid-field MRI systems has enabled the successful implementation of various CMR applications, including cardiac function and flow assessment, myocardial tissue characterization, and MRI-guided interventional procedures [4,5]. These techniques have demonstrated diagnostic image quality, and the imaging-derived cardiovascular parameters have shown agreement with those obtained from higher field MRI systems [6,7,8,9].

Among CMR applications, phase-contrast magnetic resonance (PCMR) is valuable for visualization and quantification of flow dynamics [10]. Conventional segmented k-space 2D PCMR requires electrocardiogram (ECG) synchronization, respiratory control by breath-holding over multiple heartbeats, and relies on assumptions of a regular cardiac rhythm to provide the averaged flow information over multiple cardiac cycles. However, breath-holding can be challenging for pediatric patients and for adults with cardiac or respiratory disorders, severe obesity, and claustrophobia. Additionally, segmented PCMR fails to capture the beat-to-beat variations in flow that can occur with irregular cardiac rhythm. Real-time PCMR (RT-PCMR) addresses these challenges by eliminating the need for respiratory control and regular cardiac rhythm [11], by acquiring under-sampled k-space data required for each cine frame within each heartbeat. An efficient k-space sampling strategy and a high acceleration rate are utilized to maintain adequate spatial and temporal resolutions.

Fast k-space acquisition methods such as echo-planar imaging (EPI) [12] and spiral imaging [13,14] along with advanced reconstruction techniques such as model-based reconstruction [11], compressed-sensing (CS) [15], and deep learning [16], have enabled improved acquisition efficiency and higher temporal resolution for RT-PCMR on higher field strength systems. Although spiral k-space trajectories use a given gradient system more efficiently and are motion-robust compared to EPI, sensitivity to off-resonance from B0 field inhomogeneities limits their application at higher field strengths [17]. The increased homogeneity at lower field strengths supports the utilization of longer spiral readouts, making efficient spiral k-space sampling a potentially attractive choice for RT-PCMR at mid-field. Moreover, a SENSE-based CS reconstruction of under-sampled spiral data can result in faster image acquisition and boost SNR.

Taking these factors into consideration, we propose an RT-PCMR technique using a spiral readout and inline SENSE-based [18] CS reconstruction for a commercial 80 cm bore whole-body 0.55T system (MAGNETOM Free.Max, Siemens Healthineers, Forchheim, Germany), equipped with gradients having maximum amplitude of 26 mT/m and slew rate of 45 T/m/s. Techniques such as real-time balanced SSFP cine imaging using self-gated spiral or Cartesian sampling patterns, and free-breathing 2D pseudo-golden-angle spiral PCMRI with SENSE reconstruction, have been demonstrated on prototype 0.55T systems with higher gradient performance [6,19,20]. However, the need to acquire both flow-compensated and flow-encoded acquisitions can make RT-PCMR particularly challenging on a system with limited gradients, as the longer repetition times (TR) can lead to inefficient acquisition. McGrath et al. demonstrated a self-gated, free-breathing PC-bSSFP approach using tiny golden angle radial acquisitions on the same 0.55T scanner as in our study; however, retrospective respiratory and cardiac binning was still required and thus beat-to-beat flow information was not preserved [7].

The main aim of this study was to successfully implement the proposed RT-PCMR sequence on a 0.55T system. Additionally, we sought to evaluate the feasibility and performance of this sequence for flow assessment at 0.55T, using breath-held segmented Cartesian 2D PCMR as the reference standard [21], in a small cohort of healthy volunteers and patients with cardiovascular disease.

## 2. Methods

The study was approved by the local Institutional Review Board. Healthy volunteers and patients referred to clinical cardiac MRI were prospectively recruited and gave written informed consent.

### 2.1. RT-PCMR Acquisition

**Figure 1:**
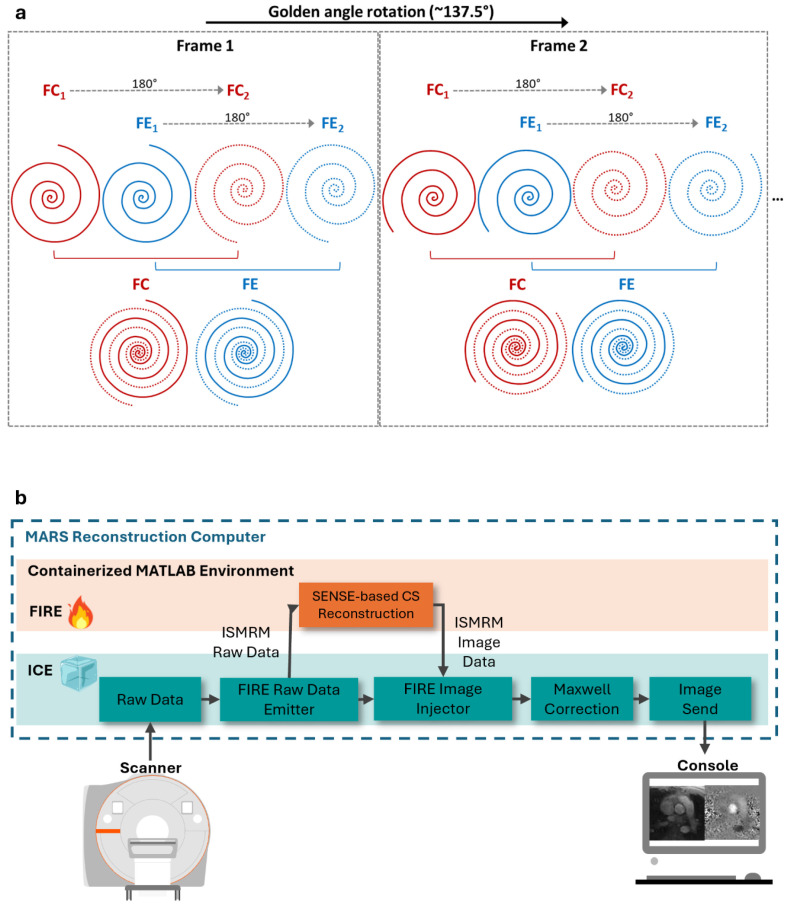
(**a**) Spiral readout acquisition: Four spiral interleaves were acquired for each cardiac frame, two for flow-compensated (FC) (red) and two for flow-encoded (FE) (blue) readouts. Within each frame, the 2nd FC and FE interleave was rotated by 180° relative to the first one to maximize the k-space coverage in each frame. From one frame to the next, the spiral readouts were continuously rotated by the golden angle (~137.5°). (**b**) Flow chart of inline spiral real-time phase contrast (RT-PC) reconstruction pipeline using Framework for Image Reconstruction Environments (FIRE). Spiral RT-PC raw data were sent using the FIRE raw data emitter from the Image Calculation Environment (ICE) to a FIRE containerized Matrix Laboratory (MATLAB) environment running on an external server for image reconstruction. The reconstructed results were then sent back to ICE using the FIRE image injector and passed through a Maxwell correction functor. The resulting images are written back to the scanner image database in Digital Imaging and Communications in Medicine (DICOM) format where they can be immediately viewed by the operator.

The 2D free-breathing spiral RT-PCMR research sequence was implemented using a dual-density spiral-out trajectory [22] with the central 30% of the spiral readout sampled at twice the Nyquist rate, and the remaining 70% sampled at 60% of the Nyquist rate (Figure 1a). Flow-compensated (FC) and flow-encoded readouts (FE) were acquired back-to-back with the same readout trajectory. Two spiral interleaves were acquired each for FC and FE within the same cardiac frame, with the second one rotated by 180° counterclockwise relative to the first. This led to a total of four spiral interleaves for each cardiac phase, with a TR of 11 ms, and a temporal resolution of 44 ms. Within each TR, the spiral readout duration was 5.32 ms. From one cardiac frame to the next, the spiral readouts were continuously rotated by the golden angle (~137.5°) [23]. The scan time was set to four seconds for each RT-PCMR image acquisition to capture at least three to four complete cardiac cycles and one full respiratory cycle in most participants.

### 2.2. RT-PCMR Reconstruction

RT-PCMR image reconstruction was implemented inline using the product reconstruction pipeline Image Calculation Environment (ICE) and Framework for Image Reconstruction Environments (FIRE) research application from Siemens (Figure 1b). The FIRE framework provides an open interface between ICE and third-party reconstruction algorithms using the International Society for Magnetic Resonance in Medicine (ISMRM) raw and image data (MRD) format [24]. Spiral RT-PCMR raw data were sent using the FIRE raw data emitter from ICE to a FIRE containerized Matrix Laboratory (MATLAB) (The MathWorks, Natick, MA, USA) environment running on an external server with a graphics processing unit (GeForce RTX 3090, 24 GB VRAM, NVIDIA, Santa Clara, CA, USA). SENSE-based CS was employed for RT-PCMR image reconstruction, with non-decimated wavelet transforms (NWT) along both temporal and spatial dimensions as the sparsity transformation. The regularization parameters were manually tuned using a single dataset to visually achieve the best image quality and then fixed for all other subjects. The density compensation function was calculated and 2D NUFFT was implemented following previously described methods [25,26]. The coil sensitivity maps were estimated from the time-averaged flow-compensated image using ESPIRiT [27]. The reconstructed results were sent back to ICE using the FIRE injector, then passed through the Maxwell correction functor implemented in ICE, and finally viewed directly on the scanner in Digital Imaging and Communications in Medicine (DICOM) format. The reconstruction time was approximately one minute for a single-slice, 90 frame, four second scan.

### 2.3. Data Acquisition

All participants were imaged on the 0.55T system. An anteriorly positioned 12-element body array coil combined with a posteriorly positioned six-element body array coil was used to receive the MR signal. External ECG gating was achieved using an MR-compatible patient monitoring device (Expression MR400, Philips N.V., Amsterdam, The Netherlands). Both free-breathing spiral RT-PCMR and Cartesian BH-PCMR research sequences were acquired perpendicular to the ascending aorta at the sinotubular junction and above the pulmonic valve in the main pulmonary artery (MPA) in each study participant. The BH-PCMR sequence, acquired with GRAPPA acceleration, served as the reference standard for comparison. The velocity encoding (VENC) was set within a difference of 50 cm/s between BH-PCMR and RT-PCMR in both vessels for all acquisitions. The acquisition parameters are described in Table 1.

### 2.4. Study Population

Adult patients aged ≥18 years with a regular cardiac rhythm and the ability to comply with scan instructions were included in the quantitative flow analysis. Exclusion criteria included standard institutional policies on contraindications to MRI, unstable clinical status, non–MRI-conditional implanted devices, and pregnancy. The study population consisted of healthy volunteers (*n* = 17) and patients with cardiovascular indications (*n* = 17). One patient was excluded from the quantitative comparison due to significant arrhythmia. MPA images were not acquired in three volunteers and one patient, resulting in 17 aortic and 14 MPA measurements in the volunteer cohort and 16 aortic and 15 MPA measurements in the patient cohort available for comparison.

### 2.5. Data Analysis

For qualitative assessment, a blur metric, with values ranging from 0 (sharp) to 1 (blur) [28], was used to quantify image sharpness. Two-dimensional flow analysis was performed in suiteHEART software (version 5.1.2, NeoSoft LLC, Pewaukee, WI, USA). Automatically generated cross-sectional vessel contours were visually assessed and manually adjusted by a reader as needed for each cardiac phase. Any phase aliasing artifacts were mitigated with phase unwrapping during post-processing [29]. Hemodynamic parameters including the net flow, peak flow rate, and peak velocity in the aorta and MPA, and pulmonary–systemic flow ratio (Qp/Qs) were determined for all individuals.

The coefficient of variation (CV), defined as the ratio of the standard deviation to the mean measurement, was assessed to determine beat-to-beat variability in RT-PCMR measurements. For quantitative comparison to BH-PCMR, RT-PCMR flow parameters were averaged for all complete heartbeats in each individual. In addition, flow parameters were compared against BH-PCMR results from 3T in a subset of patients (*n* = 6) (MAGNETOM Vida, Siemens Healthineers, Forchheim, Germany).

### 2.6. Statistical Analysis

All statistical analyses were performed using SPSS (version 29.0, Statistical Package for the Social Sciences, International Business Machines, Inc., Armonk, NY, USA) and MATLAB (version R2023a). A paired samples *t*-test was used to compare the two acquisitions. Agreement between real-time (RT) and breath-hold (BH) methods was assessed using Bland–Altman analysis and intraclass correlation coefficient (ICC). ICC > 0.9 was considered excellent agreement, 0.6–0.9 good and <0.6 moderate agreement. Bland–Altman plots were generated to compare the flow parameters between the two methods, and a reliability analysis was performed using ICC. Additionally, mean absolute error (MAE) was calculated for each of the parameters. The coefficient of variation (CV) was calculated to assess beat-to-beat variability in RR interval and flow parameters from the RT acquisition. A *p*-value < 0.05 was considered statistically significant.

## 3. Results

Compared to healthy volunteers (*n* = 17, 9 females), the patient population (*n* = 16, 11 females) was older (mean age = 45.9 ± 17.4 years vs. 30.4 ± 14.6 years, *p* = 0.01) and had a higher BMI (33.1 ± 13.4 kg/m^2^ vs. 24.1 ± 4.2 kg/m^2^, *p* = 0.02). Nine of the patients underwent CMR assessment at 0.55T as they could not be accommodated on a 70 cm bore higher field system due to obesity and/or claustrophobia concerns. The other seven patients were recruited for a dedicated research exam. Four participants (two volunteers and two patients) had Class I obesity (BMI 30 kg/m^2^–34.9 kg/m^2^), and five patients had Class III obesity (BMI ≥ 40 kg/m^2^). The patient demographics, risk factors, and MRI indications are summarized in Table 2.

BH-PCMR images were slightly sharper compared to RT-PCMR based on blur analysis. Mean blur metric values were 0.28 ± 0.03 for RT-PCMR and 0.26 ± 0.05 for BH-PCMR images for the aorta, yielding a small absolute difference of 0.018 (95% CI: 0.002–0.034; *p* = 0.03). In the MPA, RT-PCMR blur values were 0.28 ± 0.04 compared with 0.25 ± 0.04 for BH, corresponding to an absolute difference of 0.028 (95% CI: 0.011–0.045; *p* = 0.002). Although the results were statistically significant, these differences were nominal in magnitude, demonstrating adequate image sharpness for reliable analysis.

RR-interval variability in RT PCMR, measured by the coefficient of variation (CV), was slightly higher in patients than in volunteers for both aorta (5.8 ± 4.5% vs. 5.1 ± 4.7%) and MPA (5.3 ± 6.4% vs. 4.7 ± 3.2%); however, these differences were not statistically significant (aorta: *p* = 0.744; MPA: *p* = 0.635). Aortic net flow variability was significantly higher in patients than in volunteers (6.9 ± 4.2% vs. 4.3 ± 2.2%, *p* = 0.035), and also higher in the MPA but did not reach statistical significance (8.7 ± 10.1% vs. 5.1 ± 2.8%, *p* = 0.208). A similar pattern was observed for peak flow rates, with patients exhibiting significantly higher variability in the aorta (4.9 ± 3.1% vs. 2.8 ± 1.7%, *p* = 0.019), while variability in the MPA was higher but not statistically significant (5.3 ± 5.3% vs. 3.8 ± 1.6%, *p* = 0.315). Peak velocity in patients also demonstrated higher variability than volunteers in both the aorta (8.0 ± 7.5% vs. 5.4 ± 2.5%) and the MPA (8.3 ± 7.5% vs. 5.7 ± 2.1%); however, these differences were not statistically significant (aorta: *p* = 0.192; MPA: *p* = 0.219).

**Figure 2:**
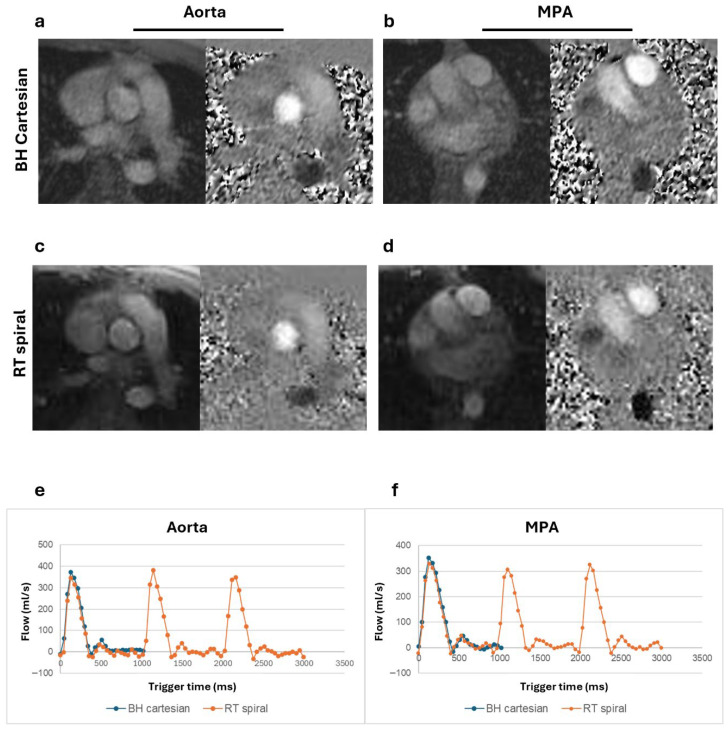
Magnitude and phase images and flow curves from a healthy volunteer (BMI = 21.0 kg/m^2^). Magnitude and phase images of the aorta and main pulmonary artery (MPA) for the breath-held (BH) Cartesian (**a**,**b**) and real-time (RT) spiral acquisitions (**c**,**d**). Total flow from the BH segmented acquisition plotted alongside the multiple complete beats from the RT spiral acquisition for both the aorta (**e**) and the MPA (**f**) show comparable flow peaks.

**Figure 3:**
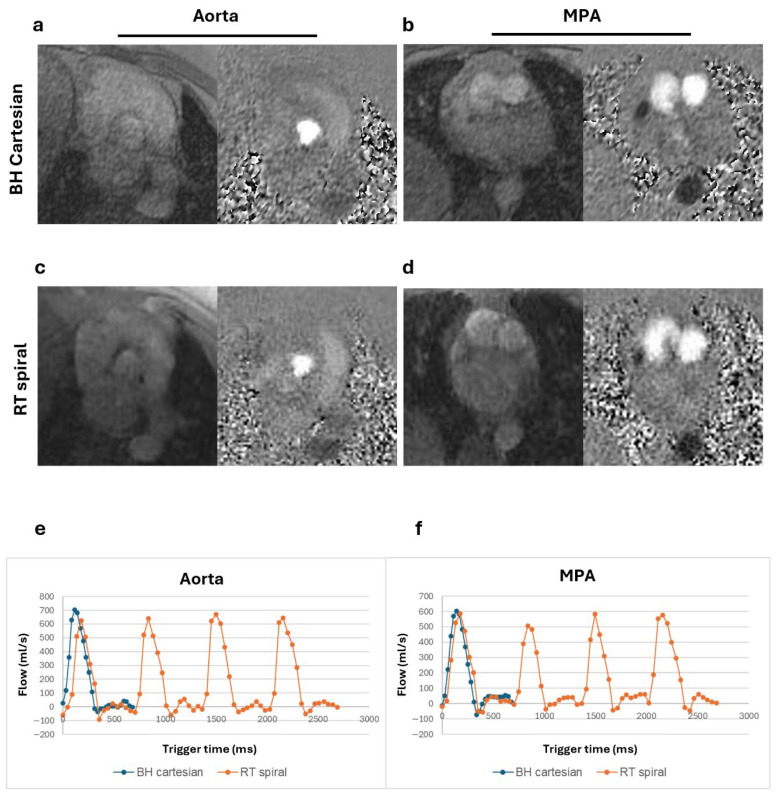
Magnitude and phase images and flow curves from a patient with suspected hypertrophic cardiomyopathy and class III obesity (BMI = 52.8 kg/m^2^). Magnitude and phase images of the aorta and main pulmonary artery (MPA) for the breath-held (BH) Cartesian (**a**,**b**) and real-time (RT) spiral acquisitions (**c**,**d**). Total flow from the BH segmented acquisition plotted alongside the multiple complete beats from the RT spiral acquisition for both the aorta (**e**) and the MPA (**f**) show comparable peaks. In comparison to Figure 2, the magnitude images are visually noisier with the larger body habitus yet provide reliable flow assessment.

**Figure 4:**
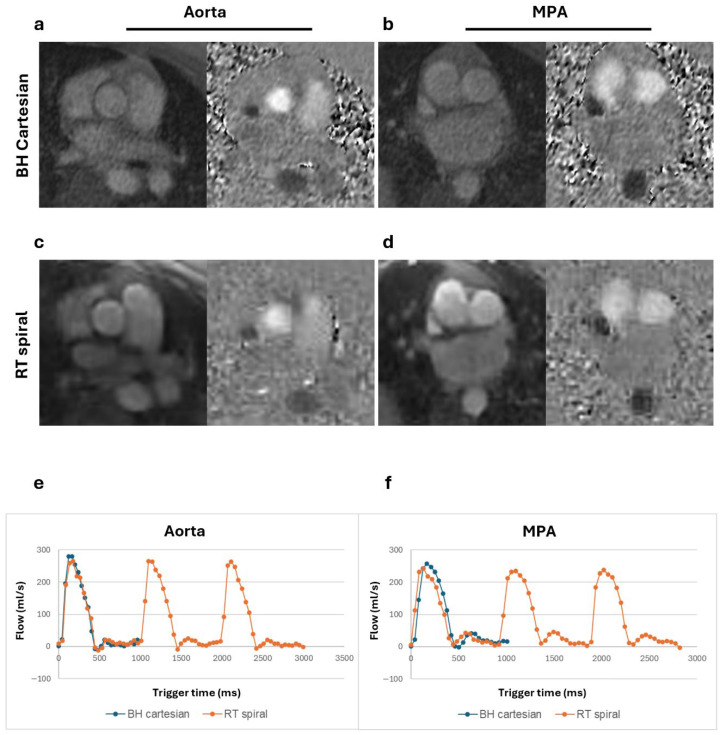
Magnitude and phase images and flow curves from an overweight patient (BMI = 26.3 kg/m^2^) with claustrophobia concerns, evaluated for chest pain and dyspnea on exertion. Magnitude and phase images of the aorta and main pulmonary artery (MPA) for the breath-held (BH) Cartesian (**a**,**b**) and real-time (RT) spiral acquisitions (**c**,**d**). Total flow curves of the aorta (**e**) and MPA (**f**) assessed from BH segmented and RT spiral acquisitions show comparable peaks.

Magnitude and phase images and corresponding flow curves of the aorta and MPA acquired from a healthy volunteer, a patient with Class III obesity, and a patient with claustrophobia are shown in Figure 2, Figure 3, and Figure 4, respectively. Similar peak flow profiles for the individual beats of RT acquisition are shown compared to the BH acquisition in all cases, underscoring the diagnostic equivalence of the two techniques for normal cardiac rhythm.

**Figure 5:**
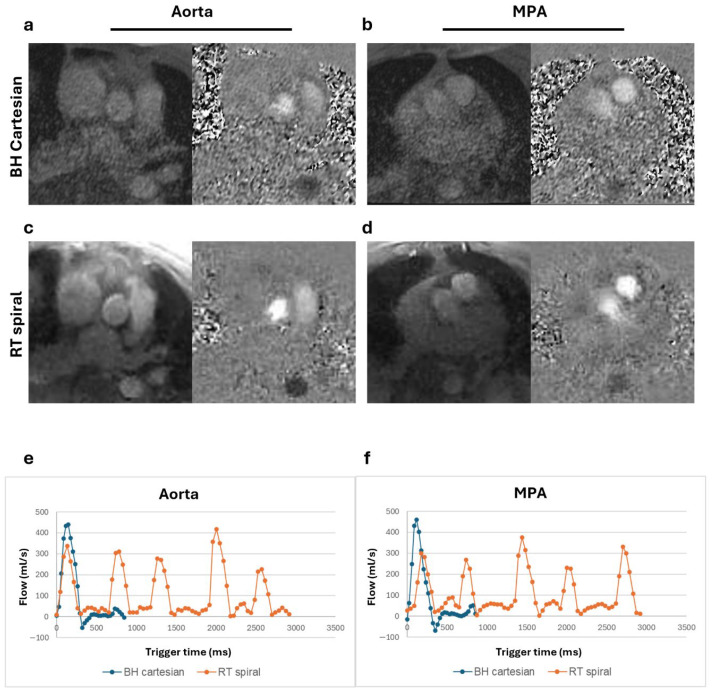
Magnitude and phase contrast images and flow curves from an arrhythmia patient with class I obesity (BMI = 32.3 kg/m^2^) Magnitude and phase images of the aorta and main pulmonary artery for the breath-held (BH) Cartesian (**a**,**b**) and real-time (RT) spiral (**c**,**d**) acquisitions. Total flow from the RT-spiral acquisition demonstrates beat-to-beat flow variability caused by arrhythmogenic activity, not characterized with the BH Cartesian acquisition (**e**,**f**). Underestimation of aortic flow by the BH acquisition is visually observed in (**e**).

PCMR images of the aorta and MPA and corresponding flow curves from a patient with arrhythmia (Figure 5), who was excluded from quantitative comparison, demonstrate that RT-PCMR was able to capture the beat-to-beat variations while BH-PCMR, reflecting the average flow of multiple heartbeats, underestimates the flow.

Table 3 summarizes the comparison of RT-PCMR and BH-PCMR quantitative flow parameters in volunteers and patients, respectively.

**Figure 6:**
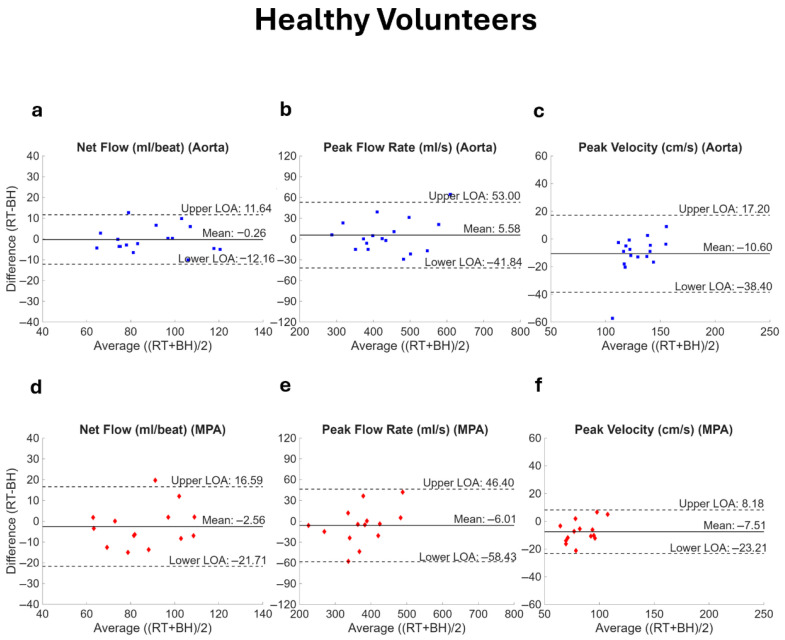
Bland–Altman plots comparing flow parameters in healthy volunteers. Net flow, peak flow rate, and peak velocity acquired with real-time (RT) spiral and breath-held (BH) Cartesian PCMR are compared for both the aorta (**a**–**c**) and the main pulmonary artery (MPA, (**d**–**f**)). The RT measurement represents the value averaged over the number of complete heartbeats available. The solid line represents the bias while the dotted lines show the limits of agreement (LOA, mean ± 1.96 standard deviation).

Bland–Altman analysis showed good agreement of RT-PCMR with BH-PCMR for both aorta and the MPA in healthy volunteers (Figure 6). There was no significant bias in net flow (aorta, *p* = 0.863; MPA, *p* = 0.344) and peak flow rate (aorta, *p* = 0.356; MPA, *p* = 0.415) between the two techniques. There was a significant bias in peak velocity (aorta, *p* = 0.001; MPA, *p* = 0.004), with RT-PCMR underestimating peak velocity in the aorta by 7.9% (MAE = 12.0 cm/s) and in the MPA by 8.6% (MAE = 9.4 cm/s). No significant bias was observed in the Qp/Qs ratio (bias = −0.04; LOA: −0.22 to 0.14; *p* = 0.087). ICC analysis demonstrated excellent agreement for net flow and peak flow rate in the aorta and MPA (ICC > 0.90, *p* < 0.001 for all). Peak velocity measurements demonstrated good agreement in the aorta and MPA (ICC > 0.70, *p* < 0.001) while the Qp/Qs ratio exhibited moderate reliability in volunteers (ICC 0.56, *p* = 0.056).

**Figure 7:**
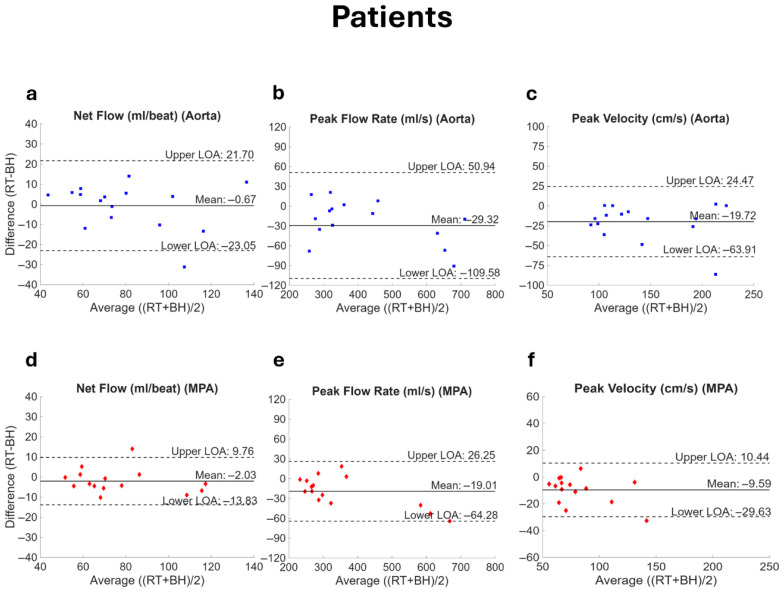
Bland–Altman plots comparing flow parameters in patients. Net flow, peak flow rate, and peak velocity acquired with real-time (RT) spiral and breath-held (BH) Cartesian PCMR are compared for both the aorta (**a**–**c**) and the main pulmonary artery (MPA, (**d**–**f**)). The RT measurement represents the value averaged over the number of complete heartbeats available. The solid line represents the mean while the dotted lines show the limits of agreement (LOA, mean ± 1.96 standard deviation).

Bland–Altman plots for flow parameters in the patient cohort are shown in Figure 7. Like the volunteers, there was no significant bias in net flow (aorta, *p* = 0.816; MPA, *p* = 0.212). Bias in peak flow rate was significant (aorta, *p* = 0.012; MPA, *p* = 0.007) with RT-PCMR slightly underestimating flow rate in the aorta by 6.2% (MAE = 35.4 mL/s) and in the MPA by 4.6% (MAE = 23.0 mL/s). The bias was also significant for peak velocity (aorta, *p* = 0.003; MPA, *p* = 0.003) with RT-PCMR underestimating in the aorta by 12.7% (MAE = 20.2 cm/s) and in the MPA by 10.4% (MAE = 10.5 cm/s). No significant bias was observed in the Qp/Qs ratio (bias = −0.02; LOA: −0.28 to 0.23; *p* = 0.592). The reliability analysis demonstrated excellent agreement for net flow, peak flow rate, and peak velocity (ICC > 0.90, *p* < 0.001 for all) and moderate reliability for Qp/Qs (ICC = 0.71, *p* = 0.015).

Comparison of RT-PCMR at 0.55T to BH-PCMR at 3T in the subset of six patients that underwent MRI scans at both systems demonstrated no significant bias in flow parameters. For the aorta, net flow was 60.1 ± 11.3 mL/beat at 3T and 70.5 ± 15.6 mL/beat at 0.55T (*p* = 0.103), peak flow rate was 325.8 ± 29.7 vs. 339.7 ± 6.4 mL/s (*p* = 0.430), and peak velocity was 106.2 ± 14.7 vs. 104.0 ± 17.5 cm/s (*p* = 0.753). In the MPA, net flow was 63.7 ± 13.2 mL/beat vs. 64.5 ± 12.9 mL/beat (*p* = 0.868), peak flow rate was 291.5 ± 52.5 vs. 284.2 ± 46.5 mL/s (*p* = 0.493), and peak velocity was 72.0 ± 10.9 vs. 67.2 ± 10.5 cm/s (*p* = 0.108). Qp/Qs differed slightly between two field strengths (1.06 ± 0.03 vs. 0.92 ± 0.08, *p* = 0.008); however, the ratios were within the physiological normal range.

## 4. Discussion

In this study, we demonstrated the feasibility of a free-breathing spiral RT-PCMR acquisition using a golden angle sampling scheme and FIRE-based CS inline reconstruction on a wide-bore 0.55T clinical MR system with moderate gradient performance. Quantitative flow parameters measured by the proposed RT-PCMR sequence were compared with a BH-segmented Cartesian PCMR acquisition [21]. The results revealed that net flow in the aorta and MPA, as well as the Qp/Qs ratio, showed good agreement with BH-PCMR while peak velocity was slightly underestimated in both patients and volunteers. Qualitative analysis showed that RT-PCMR images were more blurred than BH-PCMR, likely due to the lower spatial resolution. The differences were not substantial and did not impact the auto segmentation performance of the post-processing software overall; however, all vessel contours were manually reviewed and edited as needed on a frame-by-frame basis to ensure accuracy of flow analysis.

Free-breathing RT-PCMR eliminates the need for regular cardiac rhythm over multiple heartbeats and breath-holding, unlike segmented PCMR. Although most RT-PCMR studies have been at 1.5T [30,31] and 3T [32,33], current advances in acquisition and reconstruction methods allow the recovery of diagnostic-quality images from low SNR data and can be utilized to achieve diagnostic imaging at lower field strengths. Daude et al. used a free-breathing 2D pseudo-golden-angle PCMR sequence on the commercial 0.55T system with prototype faster gradients to develop an inline automatic quality control method that scanned until a predetermined SNR criterion was met per subject [20]. This technique provided consistent image quality based on a generalizable closed-loop feedback framework between acquisition and reconstruction. Xiang et al. demonstrated bSSFP-based 2D Cartesian BH-PCMR on a ramped down 0.55T system with high performance gradients, where the intrinsically higher SNR by bSSFP demonstrated improved image quality and similar flow results to GRE-based PCMR [34]. Both techniques use segmented acquisitions and did not resolve beat-to-beat flow.

Given the challenges of inherently lower SNR at 0.55T and the moderate gradient performance of the commercial system used in this study, we chose to implement a dual-density spiral readout RT-PCMR sequence with CS reconstruction. Compared to uniform Cartesian sampling patterns, variable density sampling and non-Cartesian trajectories provide more efficient k-space coverage, leading to faster scan times. The current SCMR guidelines recommend a temporal resolution under 50 ms for PCMR, with one study suggesting an optimal temporal resolution of approximately 40 ms [35,36]. Meeting these requirements with RT-PCMR is particularly challenging due to the need to acquire both flow-compensated and flow-encoded acquisition within each cardiac phase. Despite the longer TR necessitated by the system’s gradient performance compared to standard high-field and prototype mid-field systems, we achieved a temporal resolution of 44 ms, using only four spiral arms per frame (two for flow compensation and two for flow encoding), by leveraging the efficiency of spiral k-space sampling and CS-SENSE reconstruction, and by slightly sacrificing spatial resolution compared to the BH-PCMR protocol.

Our attempts to implement Cartesian RT-PCMR on this system were not successful, since fewer than four k-space lines (given TR = 6.7 ms in Cartesian PCMR) could be acquired per FC and FE frame to reach a temporal resolution of 44 ms as could be achieved with spiral RT-PCMR. To do so would lead to a 20- to 30-fold acceleration, which is overly aggressive and would impact image quality and flow quantification at 0.55T. Spiral acquisition covers a larger k-space per RF excitation compared to Cartesian sampling, requiring approximately 5–10 times fewer readouts and therefore shortening the overall scan duration. The benefits of a spiral readout can hence be fully utilized on a mid-field system, enabling high-quality real-time imaging. Further SNR efficiency improvements may be possible by optimizing the spiral trajectory as described by Kowalik et al [15].

CS reconstruction methods speed up image acquisition but are often limited by the computational complexity, long reconstruction times, and extensive offline construction, limiting their clinical application. The FIRE research prototype for inline reconstruction used in this study offers flexibility to utilize an external GPU-equipped server to speed up image reconstruction and to deliver images to the scanner in under a minute. This setup benefits the clinical workflow, as image reconstruction can be performed in the background while subsequent imaging protocols are acquired, providing images back to the operator and reader in a timely manner for interpretation. Recently, Haji-Valizadeh et al. developed a complex difference DL network and showed a 4.6-fold faster reconstruction time compared to CS [16]. Similarly, Jaubert et al. extended the work of Kowalik et al. by proposing a DL artifact suppression model for perturbed spiral trajectories on a 1.5T system, achieving a reconstruction speed 15 times faster than CS reconstruction [15,37]. Implementing DL-based approaches to our technique on 0.55T and the utilization of advanced computing hardware may further improve reconstruction speed, image display and clinical integration. However, this was not explored as the aim of this study was not to achieve true real-time image display during data acquisition to visualize the images while they are acquired, but rather to implement and evaluate a real-time flow acquisition in which each image frame is obtained from a single rapid acquisition (~40 ms), without k-space segmentation across multiple heartbeats, enabling clinically feasible reconstruction for quantitative flow assessment.

A major clinical advantage of RT-PCMR is its ability to preserve beat-to-beat flow information which allows the evaluation of physiological (e.g., respiratory effects) and pathological (e.g., arrhythmia) blood flow variations. RT-PCMR has been able to characterize respiratory cycle-dependent flow variations across mitral and tricuspid valves in a patient cohort with constrictive pericarditis [38]. RT-PCMR has also been demonstrated in atrial fibrillation patients at 1.5T and the detection of beat-to-beat variability with RT-PCMR has previously been compared to 4D flow results [39]. RR intervals were relatively consistent over the imaging duration and a low coefficient of variability (~5%) was demonstrated for flow parameters across the multiple heartbeats in this cohort of healthy volunteers and patients, consistent with the absence of significant arrhythmia. Notably, in the anecdotal example in a patient with atrial fibrillation, the flow curves illustrated that RT-PCMR successfully captured the beat-to-beat fluctuations in flow dynamics. Additional systematic evaluations in larger patient cohorts with arrhythmia are warranted for further validation.

While RT-PCMR net flow and peak flow rate agreed with BH-PCMR for both vessels and across volunteers and patients, we observed an underestimation in peak velocity. This was greater in patients (13% in the aorta and 10% in the MPA) than volunteers (approximately 8% in both the aorta and MPA). The underestimation observed with RT-PCMR likely stems from a combination of technical factors such as lower SNR at 0.55T, higher acceleration rates, spatial blurring introduced by lower spatial resolution, and spatiotemporal smoothing from regularization applied in CS reconstruction [40]. Deep learning (DL)-based reconstruction [16] could be investigated in the future to further speed up image acquisition and improve spatial resolution.

Physiological factors such as a breath-holding vs. free-breathing state can contribute to peak velocity underestimation; measurements during breath-holds tend to be higher than free-breathing RT-PCMR due to more consistent cardiac filling and reduced respiratory motion [41]. The relatively higher underestimation in the patient cohort may likely be due to body habitus related imaging conditions, as they had a higher mean BMI than volunteers. The increased distance between the receiver coils and cardiac structures can lead to diminished SNR and greater noise contamination, collectively impacting velocity estimation accuracy. According to current SCMR guidelines, aortic stenosis is classified as mild when peak aortic jet velocity is 2.0–2.9 m/s, moderate at 3.0–3.9 m/s, and severe when peak velocity exceeds 4.0 m/s. Similarly, a peak velocity of >4 m/s is used to classify pulmonic stenosis [42]. Within this clinical framework, the degree of peak velocity underestimation observed with RT-PCMR in the present study was small and remained within clinically acceptable limits, suggesting that it is unlikely to affect stenosis severity classification. The Qp/Qs ratio demonstrated no significant bias but moderate ICC reliability in this cohort without intracardiac shunts and therefore having a narrow range of Qp/Qs values; the lower reliability may be due to the nature of Qp/Qs being a ratio, thereby amplifying small variations in pulmonary or systemic flow measurements.

In a small subset of patients scanned at both field strengths, RT-PCMR at 0.55T showed overall agreement with BH-PCMR at 3T for major flow parameters, with no significant differences in aortic or MPA net flow, peak flow rate, or peak velocity. Although Qp/Qs differed modestly, values remained within the physiological normal range. Given the limited number of paired examinations (*n* = 6), these findings should be interpreted as preliminary, but they nonetheless support the feasibility of RT-PCMR at 0.55T for quantitative flow assessment and comparability to measurements at a higher field strength.

Mid-field MRI systems are being explored as an appealing alternative to higher-field strength scanners due to their reduced space needs and lower purchase price and maintenance costs [2]. The wider bore in this study can better accommodate patients with higher BMI, anxiety, and claustrophobia, compared to 60–70 cm bore systems. The reduced field strength may also benefit patients with metallic implants due to lower device overheating risk [43], and those with large body habitus due to reduced SAR. In our study, nine patients could not be comfortably accommodated in a 70 cm bore but were successfully assessed with the 80 cm bore 0.55T system. Though images for obese participants were visually noisier, likely due to greater distance between the receiver coils and region of interest, the RT-PCMR flow results matched those of standard segmented k-space BH acquisition.

## 5. Study Limitations

This initial validation study is limited by the small sample size in an adult population, thereby limiting the statistical power and generalizability of conclusions. The technique needs to be further assessed in the pediatric population where the spatiotemporal requirements are higher due to the higher heart rates and smaller body habitus. Another limitation of this study is the absence of flow phantom validation; however, a previously validated breath-held segmented acquisition was used as an in vivo reference standard for comparison [21]. A dual-density spiral readout was utilized to facilitate an incoherent sampling pattern to support efficient CS-SENSE reconstruction, but the potential of a perturbed spiral trajectory together with CS reconstruction, as demonstrated by Kowalik et al. [15], could be explored to further optimize efficacy and measurement accuracy, including peak velocity. Spiral deblurring algorithms have been shown to enhance image quality but were not used in this study. The regularization parameters for the CS reconstruction were tuned using a single reference dataset. Although the selected parameters produced consistent image quality across all data acquisitions and were deemed satisfactory for the purposes of this feasibility study, a more comprehensive sensitivity analysis would be valuable and is warranted in future work, but was outside the scope of the present study. A larger flip angle was utilized for RT-PCMR to account for the longer TR compared to BH-PCMR, but further parameter optimization is warranted. There were no patients with significant arrhythmias, intra-cardiac shunting or regurgitant flow to further assess RT-PCMR performance for these clinical indications.

## 6. Conclusions

In conclusion, we implemented and validated the feasibility of a free-breathing golden-angle spiral RT-PCMR sequence on a commercial wide bore 0.55T MR system with moderate gradient performance in a preliminary cohort of healthy volunteers and patients. The application provides a promising tool when free-breathing, real-time flow assessment is desired in obese and claustrophobic patient cohorts who cannot be evaluated on narrower-bore higher-field systems. Therefore, free-breathing RT-PCMR imaging on this scanner can further contribute to increased CMR access for obese and claustrophobic patients.

## Figures and Tables

**Table 1:** Image acquisition parameters for breath-held and real-time phase contrast images.

Parameters	Breath-Held Cartesian	Real-Time Spiral
TE/TR (ms)	3.71/6.7	3/11
FOV (Aorta) (mm × mm)	(240–420) × (225 × 436)	300 × 300
FOV (MPA) (mm × mm)	(262–436) × (225 × 440)	300 × 300
Spatial resolution (Aorta) (mm × mm)	(1.2–2.0) × (1.7–3.4)	2.3 × 2.3
Spatial resolution (MPA) (mm × mm)	(1.3–2.1) × (1.7–3.1)	2.3 × 2.3
Temporal resolution (ms)	53	44
Slice thickness (mm)	8	8
Acceleration factor (R)	2	6
Bandwidth (Hz/Px)	401	1116
Flip angle (degrees)	12	20
Acquisition time	10 heart beats	4 s
VENC Aorta (cm/s)	200–250	150–250
VENC MPA (cm/s)	150–200	150–200

Abbreviations: TE: echo time, TR: repetition time, FOV: field of view, VENC: velocity encoding, and MPA: main pulmonary artery.

**Table 2:** Demographics, risk factors, and clinical indications for patients undergoing cardiac MRI.

Patient	Age (Years)	Sex	BMI (kg/m^2^)	Indications for CMR Evaluation
1	65	F	26.3	Chest pain and dyspnea on exertion.
2	22	M	28.2	Multiple arrythmias. History of supraventricular tachycardia, NSVT
3	51	F	16.8	Suspected cardiac dysfunction in the setting of PVCs, inconclusive echocardiogram.
4	21	F	29.2	Left ventricular dysfunction, abnormal echocardiogram.
5	48	F	24.3	Regional wall motion abnormalities.
6	32	F	18.6	NSVT.
* 7	67	M	32.3	Renal amyloidosis.
8	19	F	27	Suspected bicuspid aortic valve.
9	57	F	25.2	Serial aortic surveillance, Takotsubo cardiomyopathy patient.
10	47	M	52.8	Hypertrophic cardiomyopathy, abnormal resting echocardiogram.
11	34	M	48.8	Suspected dilated aortic root and ascending aorta.
12	55	M	29.5	Cardiac viability in the setting of multiple comorbidities, claustrophobic.
13	60	F	64	Rule out infiltrative disease.
14	67	M	48	Suspected aortic dilation, high PVC burden present.
15	63	F	30	Evaluate aortic and mitral regurgitation, sub-aortic membrane.
16	26	F	21.3	Follow-up for hypertrophic cardiomyopathy.
17	66	F	41.9	Evaluation for left ventricular hypertrophy.

Abbreviations: NSVT: non-sustained ventricular tachycardia; PVC: premature ventricular contraction. * Patient excluded from the data analysis.

**Table 3:** Flow parameters for healthy volunteers and patients.

	BH-PCMR	RT-PCMR	ICC (95%CI)	*p*-Value (ICC)
**Healthy Volunteers**				
**Net flow (ml/beat)**				
Aorta (*n* = 17)	89.4 ± 17.8	89.2 ± 17.5	0.97 (0.92–0.99)	**<0.001**
MPA (*n* = 14)	87.7 ± 15.6	85.1 ± 17.7	0.91 (0.71–0.97)	**<0.001**
**Peak flow rate (ml/s)**				
Aorta (*n* = 17)	434.5 ± 87.0	440.1 ± 93.2	0.98 (0.95–0.99)	**<0.001**
MPA (*n* = 14)	374.4 ± 67.8	368.4 ± 77.8	0.96 (0.90–0.99)	**<0.001**
**Peak positive velocity (cm/s)**				
Aorta (*n* = 17)	134.5 ± 12.7	125.2 ± 16.6	0.79 (0.12–0.93)	**<0.001**
MPA (*n* = 14)	87.5 ± 11.9	80.0 ± 15.3	0.84 (0.21–0.96)	**<0.001**
**Qp/Qs (n = 14)**				
	1.00 ± 0.10	0.95 ± 0.08	0.56 (−0.18–0.85)	0.056
**Patients**				
**Net flow (ml/beat)**				
Aorta (*n* = 16)	80.4 ± 27.3	79.8 ± 24.4	0.95 (0.86–0.98)	**<0.001**
MPA (*n* = 15)	77.7 ± 22.4	75.7 ± 21.1	0.98 (0.94–0.99)	**<0.001**
**Peak flow rate (ml/s)**				
Aorta (*n* = 16)	433.3 ± 168.3	403.9 ± 154.6	0.98 (0.89–0.99)	**<0.001**
MPA (*n* = 15)	363.7 ± 152.1	344.7 ± 136.0	0.99 (0.94–0.99)	**<0.001**
**Peak positive velocity (cm/s)**				
Aorta (*n* = 16)	153.1 ± 50.6	133.3 ± 46.7	0.91 (0.50–0.97)	**<0.001**
MPA (*n* = 15)	86.4 ± 28.6	76.8 ± 24.3	0.93 (0.55–0.98)	**<0.001**
**Qp/Qs (n = 15)**				
	1.03 ± 0.14	1.01 ± 0.13	0.71 (0.13–0.90)	**0.015**

Bold *p*-values indicate statistical significance.

## Data Availability

The data supporting the conclusions of this article will be made available by the authors upon request.
